# Effects of nebulized dexmedetomidine for premedication on the parameters of oxidative and inflammatory stress in children undergoing tonsillotomy and adenoidectomy: A pilot randomized controlled trial

**DOI:** 10.1371/journal.pone.0348763

**Published:** 2026-05-11

**Authors:** Vesna V. Stevanovic, Ana D. Mandras, Ana A. Djuric, Ivan A. Soldatovic, Dragana V. Bojanin, Branko Lj. Lukic, Predrag D. Stevanovic, Marija M. Stevic, Aleksandar M. Vlahovic

**Affiliations:** 1 Department of Anesthesiology, Mother and Child Health Care Institute of Serbia “Dr Vukan Cupic”, Belgrade, Serbia; 2 University of Belgrade, Faculty of Medicine, Belgrade, Serbia; 3 Department for Experimental Oncology, Institute for Oncology and Radiology of Serbia, Belgrade, Serbia; 4 Institute of Medical Biostatistics, Faculty of Medicine, Belgrade, Serbia; 5 Department for Clinical Chemistry and Hematology, Mother and Child Health Care Institute of Serbia “Dr Vukan Cupic”, Belgrade, Serbia; 6 Department of general surgery, Clinical Hospital Center “Zemun”, Belgrade, Serbia; 7 Department of Anesthesiology, Clinical Hospital Center “Dr Dragisa Misovic-Dedinje”, Belgrade, Serbia; 8 Department of Anesthesiology, University Children’s Hospital, Belgrade, Serbia; 9 Department of Plastic Surgery, Mother and Child Health Care Institute of Serbia “Dr Vukan Cupic”, Belgrade, Serbia; University of Split Faculty of Medicine: Sveuciliste u Splitu Medicinski fakultet, CROATIA

## Abstract

**Background:**

This prospective randomized clinical pilot study aimed to evaluate the effect of inhaled Dexmedetomidine in premedication in children after tonsillectomy with adenoidectomy on oxidative and inflammatory stress and the drugs impact on clinical parameters such as perioperative sedation, hemodynamics, respiratory function, postoperative delirium, analgesia, opioid consumption and frequency of complications.

**Methods:**

Children aged 4–13 years with an indication for tonsillotomy with adenoidectomy were randomized into two groups: (D) – Dexmedetomidine treated group and (N) – control, non-treated group. Ramsey’s sedation score, Bispectral index (BIS), and vital signs (heart rate, respiratory rate, noninvasive blood pressure and hemoglobin oxygen saturation) were monitored perioperatively in at defined time points. After 15 minutes of extubation, the postoperative delirium score (PEAD) and pain score (VAS) were noticed. Pain scores were monitored for up to 12 postoperative hours every three hours on the ward. Venous blood samples were taken before intervention and after surgery to evaluate redox homeostasis (levels of malondialdehyde – MDA, nitrogen monoxide, glutathione and sulfhydryl groups and the activity of superoxide dismutase and catalase) and inflammatory parameters (blood count, neutrophile/lymphocyte ratio, C-reactive protein and Interleukin 6). Perioperative complications in the induction phase, intraoperative and postoperative period, as well as the consumption of opioids, were noted.

**Results:**

16 patients in the D and 15 in the N groups participated in the study. There was no difference in age, sex, body weight and duration of surgery between the two groups. The obtained results of oxidative stress and inflammatory response markers showed that there was a statistically significant difference in the MDA level between compared groups (postoperative MDA 88.93 ± 1.19 vs. 95.05 ± 1.58, ΔMDA 0.014), with no difference observed in other investigated oxidative and inflammatory stress in plasma samples. Patients in both groups were hemodynamically and respiratory stable. Still, patients in the treated (D) group achieved the desired level of sedation compared to the control evaluated by the Ramsey sedation score (p < 0.001). The BIS values were statistically significantly different between the D and N groups 30 minutes after inhalation (86.6 ± 11.6 vs. 98.1 ± 1.2, P < 0.001), but showed no differences during and after the procedure. The two groups had a significant difference in pain control upon awakening and one hour after the operation, respectively (pain on awakening p = 0.024; pain during the first hour of stay in the ward p = 0.039). The consumption of intraoperative opioids was slightly lower in the D group than in the N group (D vs. N group: fentanyl in µg/kg mean 3.67 ± 0.78 vs. 3.94 ± 1.12, p = 0.445, i.e., alfentanil in µg/kg mean 2.02 ± 1.85 vs. 2.30 ± 2.08, p = 0.691). Also, there were no differences in observed perioperative complications between groups.

**Conclusion:**

Inhaled Dexmedetomidine at a dose of 2 µg/kg in premedication reduces oxidative stress. However, the statistical significance of this drugs effect on the level of markers of antioxidant defense and inflammation has not been proven in children operated on for tonsillar hypertrophy. This dose regimen and route of application of Dexmedetomidine significantly affect the degree of perioperative sedation and BIS values, provides perioperative hemodynamic and respiratory stability, significant immediate postoperative analgesia, and reduces the consumption of analgesics with minimal complications, which makes it useful for shorter surgical interventions.

## 1. Introduction

Surgical procedures trigger a complex systemic inflammatory and neurohumoral response, as well as oxidative stress, which significantly impacts postoperative recovery and treatment outcomes [1]. Anesthesiological techniques and medications influence the intensity and consequences of those complex reactions to surgical trauma in various ways [2]. In children, the level of oxidative stress to surgical stressors is higher due to the immaturity of the antioxidant defense system and inflammatory response [3,4]. Therefore, premedication as an critical component of pediatric anesthesia. The clinical objectives of premedication include anxiolysis, analgesia, postoperative amnesia, reduction of the side effects of anesthetic agents, and a decrease in perioperative complications and infections. Premedication drugs in pediatric anesthesia can be administrated orally, buccally, nasally, rectally and intravenously [5]. Recent studies in children have shown that nebulized premedication using agents such as ketamine, midazolam, fentanyl, and Dexmedetomidine (DEX) offers clinical advantages due to their rapid onset of action and high absorption through the respiratory mucosa [6,7]. Meta-analysis of both animal and human studies has indicated that perioperative administration of the alpha 2 agonist DEX can reduce systemic and local oxidative stress, as well as the inflammatory response [8,9]. DEX also enhances positive clinical outcomes during anesthesia by modulating the neurohumoral response. It is known that DEX is a safe drug to use, but its efficacy on inflammatory and oxidative stress for nebulized administration has not been explored in the pediatric population. Thus, our research would contribute to the knowledge of whether and to what extent nebulized DEX affects the parameters of oxidative stress, antioxidant defense, and inflammatory response markers, as well as the clinical manifestations of its application. Our study will evaluate the effects and potential benefits of nebulized DEX in the perioperative treatment of pediatric patients undergoing tonsillar hypertrophy surgery. These patients are typically familiar with the use of inhalers, and tonsillotomy with adenoidectomy is a relatively brief surgical procedure. This setting allows us to investigate whether DEX might also provide a local analgesic effect, considering it can be retained at the surgical site in the epipharynx and on the tonsils through inhalation. Considering that tonsillotomy with adenoidectomy in children causes perioperative oxidative and inflammatory stress, we hypothesized that nebulized DEX as a premedication reduces markers of oxidative stress (malondialdehyde, nitric oxide, glutathione, superoxide dismutase, catalase, total sulfhydryl groups) and inflammatory markers (values of white blood cells, neutrophil to lymphocyte ratio, interleukin 6 and C-reactive protein) compared to placebo. Additionally, we evaluated sedative (Ramsay score sedation and Bispectral index) and analgesic (Visual Analogue Scale) effects from this alternative way of DEX administration with hemodynamic and respiratory stability in this pediatric group of patients. Furthermore, the administration of DEX could reduce intraoperative opioid consumption and lower incidence of postoperative delirium (The Pediatric Anesthesia Emergence Delirium – PAED) and complications.

## 2. Materials and methods

This prospective, randomized clinical trial as a pilot study included 34 patients 4–13 years old scheduled for tonsillotomy and adenoidectomy in general anesthesia. Prior to study start, approvals of from ethics committees were obtained: the Institute for Mother and Child Health Care Ethic Committee, Institute for Mother and Child Health Care “Dr. Vukan Cupic”, Belgrade, Serbia (registration number 10/25.01.2023) and the Institute for Oncology and Radiology of Serbia Ethic Committee, Belgrade, Serbia (registration number 01–1/2023/2779/27 Oct 2023) were obtained. As we subsequently decided to submit the manuscript to your journal, study is registered in the Australian New Zealand Clinical Trial Registry (ANZCTR) portal, which is part of https://www.who.int/clinical-trials-registry-platform/network/primary-registries in order to meet the criteria required by your journal (trial registration number: ACTRN12625000041459). The authors confirm that all ongoing and related trials for this drug/intervention are registered. After obtaining informed consent from parents/legal guardians, patients were recruited between 27^th^ of February and 2^nd^ of August 2023. The study was performed in compliance with the Declaration of Helsinki. Inclusion criteria were: children 4–13 years, American Association of Anesthesiologists classification (ASA classification) I-II. Exclusion criteria were cardiac, pulmonary, neurological, and renal diseases, liver damage, endocrinological diseases, allergies to drugs used in the study, obesity, long-term use of antihypertensives, corticosteroids or anti-inflammatory drugs, current or recent upper airway infection and vaccination in the past one month. In case of serious complications, patients were excluded from the study.

Thirty-four patients were randomized into two groups using a simple randomization algorithm. No masking was applied in this study. The D group received premedication by inhaling DEX (n = 17 patients) in a dose of 2 µg kg-1, dissolved in 2.5 ml of normal saline. The N group was the placebo group (n = 17 patients). Children of the N group inhaled 2.5 ml of normal saline solution.

### 2.1. Investigation protocol

The day before the surgical intervention, laboratory analyses were evaluated: complete blood count (KKS_1_), coagulation status, C reactive protein (CRP_1_), and interleukin 6 (IL6_1_). Counts of white blood cells (WBC) and the ratio of absolute neutrophil counts to absolute lymphocyte counts were calculated from KKS (NLR). As part of the preoperative preparation, advice was given on the allowed preoperative intake of food and fluids according to the European Society for Pediatric Anesthesiology (ESPA) protocols [10]. Before inhalation of DEX or placebo, all patients were monitored for sedation (Ramsey sedation score), heart rate (HR t_0_), respiratory rate (RR t_0_), systolic blood pressure (SBP t_0_), mean blood pressure (MAP t_0_), diastolic blood pressure (DBP t_0_), as well as hemoglobin oxygen saturation (SpO_2_ t_0_) and possible cardiac and respiratory complications (EDANUSA X10 Patient Monitor). Ramsey’s sedation score included: 1- awake, anxious; 2 – awake, cooperative, oriented, calm; 3 – awake, responds to commands; 4 – sleeps, responds to stimulus; 5 – sleeps, reacts slowly to stimuli; 6 – sleeps, does not respond to stimuli. A score greater than 3 meant acceptable sedation. At the same time, electrodes for monitoring the Bispectral index (BIS t_0_) were placed in the frontal area to monitor the state of the electrical activity of the brain. A 20-gauge venous cannula was introduced in the cubital vein of a patient with the use of local anesthetic cream due to the need to take study blood samples before any intervention and to perform general anesthesia. After placing the venous line, the first blood sample was taken for markers of redox status (2 ml of blood per sample): malondialdehyde – MDH_0_, nitrogen monoxide – NO_0_, GSH_0_, superoxide dismutase – SOD_0_, catalase – CAT_0_ and SH_0_. Then, the inhalation of DEX or placebo began through a mask, which had to be well adapted to the child’s face (Compressor Nebulizer, model BR-CN 171, R&B Medical Company). Inhalations lasted an average of 10–15 minutes until the mist stopped coming from the inhaler chamber. The operational air flow generated in the inhaler during inhalation was 7 ± 1 L/min, allowing about 80% of the drug particles to reach the respiratory system. 30 minutes from the start of inhalation in the induction room were monitored the Ramsey sedation score and vital signs again (t_1)_ and patients received intravenous midazolam in a dose of 0.05 mg kg-1. The patients were transported to the operating room. As part of the standard procedure, monitoring was installed (*Dräger* monitor, Germany): three-channel ECG, pulse oximetry, noninvasive blood pressure, BIS electrodes, capnography, capnometry and respiratory monitoring. Vital signs were assessed in the t_2_ time and for five minutes (t_2_ – t_x_), depending on the duration of the surgery. The duration of surgical intervention in minutes was recorded as we measured the consumption of drugs in general anesthesia in both groups of patients. Patients of both groups received Hartman solution in a 4 ml kg-1 h dose during the intervention. After preoxygenation with 100% oxygen for three minutes to introduce intravenous anesthesia, the children received atropine 0.01 mg/kg-1, propofol 2.5–3.5 mg kg-1, fentanyl 5 µg kg-1, and rocuronium 0.45–0.6 mg kg-1. Manual ventilation with 100% oxygen lasted until we had ideal conditions for intubation. Afterward, the children were mechanically ventilated with a mixture of oxygen: air 0.35:0.65, with a flow rate of 6.5 L/min (*Dräger* Perseus A500 anesthesia machine). The parameters of the volume-controlled ventilation (VCV) mode of mechanical ventilation during the intervention were: tidal volume (TV) of 6–8 ml kg-1, the respiratory rate (RR) depending on age and positive end-expiratory pressure (PEEP) of 5 cmH_2_O, as well as the ratio of inspiration and expirium 1:2, aiming for PEtCO_2_ to be between 35–45 mmHg. The value of end-tidal CO_2_ is monitored on the display of the anesthesia machine. Anesthesia was maintained by inhalation of oxygen: air mixture and intermittent boluses of propofol 0.5–2 mg kg-1 with continuous monitoring of BIS values with a target BIS value of 40–60. During the procedure, children received additional intravenous boluses of opioids – fentanyl 1.25–2.5 µg kg-1 and Alfentanyl 5–15 µg kg-1, muscle relaxant rocuronium 0.15 mg kg-1 and propofol 10–20 mg in case the BIS values exceeded 60. Intraoperatively, patients of both groups received ondansetron 0.1 mg kg-1 (max 4 mg) for the prevention of nausea and vomiting and as pain therapy paracetamol in a dose of 15 mg kg-1. After successful decurarization with neostigmine 0.05 mg kg-1 and atropine 0.01 mg kg-1 at the end of the surgery, the children were extubated in the operating room and transferred to the recovery room. After 15 minutes of extubation, venous analysis was taken (for redox status markers), vital signs (heart rate, arterial pressure values, respiratory rate, saturation, and BIS), agitation score (The Pediatric Anesthesia Emergence Delirium Scale – PEAD) and pain score (Visual Analog Scale – VAS) were recorded. The VAS scale is ranked from 0–10, where zero means the absence of pain, and ten is the most potent pain. When the Aldrete recovery score was estimated to be greater than 9, the children were transferred to the otolaryngology department. The pain score was monitored in the ward with the VAS scale for three hours over the next 12 hours. Also, the frequency and type of analgesics and postoperative complications were recorded. After 6 hours of arrival at the ward, the child’s control blood count, CRP_2_ and IL6_2_ were taken. For pain control, the children received paracetamol intravenously and ibuprofen orally. In case of breakthrough pain with VAS > 4, “rescue medicine” – tramadol 1 mg kg-1 was given. Potential complications were monitored during all three follow-up phases. Hypotension was considered a value of systolic arterial pressure lower than 20% of the basic values of arterial pressure. Hypertension was considered an increase in arterial pressure by more than 20% of the baseline values. Bradycardia was considered a heart rate value lower than the initial values by 20%, and tachycardia increased the heart rate by 20% compared to the basic values. Desaturation is defined by a SpO_2_ value lower than 92%.

### 2.2. Blood sample preparation

Plasma samples separated by centrifugation at 4000 rpm for 10 minutes at room temperature were divided into aliquots and stored at −20°C for three days. After that, they were stored at −80°C until definitive analysis.

#### 2.2.1. Blood sample preparation.

*Assay of Malondialdehyde (MDA)*: MDA content was determined by the spectrophotometric method by Vilicarra et al. [11]. First, thiobarbituric acid (TBA) reagent (15% trichloroacetic acid and 0.375% TBA water solution, Merck, Darmstadt, Germany) was mixed with plasma samples, heated to 95°C, centrifuged, and then the absorbance was measured at 532 nm. Results are expressed as the mean MDA concentration (µM/mL) ± SEM [12].

*Assay for nitric oxide determination (NO):* Supernatants were lysed by sonication and centrifuged at 15000 g for 5 min at 4°C for NO determination. Because NO is an unstable molecule, it is common to measure concentrations of its products, nitrites and nitrates. As previously described by Adzic et al., nitrates were reduced into nitrites using metallic cadmium (by Navarro-Gonzálvez et al. 1998). Then, the total nitrites were quantified directly spectrophotometrically at 492 nm using the Griess’ colorimetric method. Griess reagent consists of 1.5% sulfanilamide (Sigma-Aldrich, Munich, Germany) in 1 M HCl and 0.15% N-(1-naphthyl) ethylenediamine dihydrochloride (Fluka, Buchs, Switzerland) in deionized water. To generate a standard curve, known concentrations of sodium nitrite (Mallinckrodt Chemical Works, St. Luis, MO, USA) were used, from which the nitrite concentration in the samples was calculated. All samples were run in duplicate, in two separate determinations, and the results are expressed as the mean concentration of nitrites (μM/mL) ± SEM for each group [13].

*Assay of total glutathione (tGSH)*: Total GSH (GSH + 1/2 GSSG, in GSH equivalents) content was assessed by DTNB-GSSG reductase recycling assay. The content of tGSH was spectrophotometrically measured at 412 nm for 6 min by 5,5-dithiobis-2-nitrobenzoic acid (DTNB) – oxidized glutathione (GSSG) recyclable method. The formation rate of 5-thio-2-nitrobenzoic acid (TNBA) is proportional to the total glutathione concentration. Oxidized glutathione (GSSG) was used (50 mmol) to construct a calibration curve in the range of 0.2–1 nmol/ 25 x 10–6 mL (8–40 nmol/ mL). Based on the equation of the standard curve, the content of tGSH was calculated, and the results are expressed as nM/mL ± SEM [14].

*Assay of total superoxide dismutase activity (tSOD)*: The activity of tSOD (EC 1.15.1.1.) was measured spectrophotometrically by determining a decrease in the rate of the spontaneous epinephrine autoxidation in alkaline pH at 480 nm. The kinetics of enzyme activity was measured in 0.1 mM EDTA and 50 mM carbonate buffer pH 10.2 (Serva, Feinbiochemica, Heidelberg, New York), after the addition of 10 mM epinephrine (Sigma, St. Louis, (USA)) as previously described by Djukic 2012. and Sun and Zigman 1978 [15]. The results are presented as units per mg of total protein (U/mg protein), where one unit is the enzyme required for 50% inhibition of autoxidation of epinephrine.

*Assay of catalase activity (CAT):* Catalase activity was assessed by a method based on the spectrophotometric determination of the colored complex formed between ammonium molybdate and H_2_O_2_ at 405 nm. The results are presented as units per milligram of total protein (U/mg protein), though the unit represents an amount of H_2_O_2_ reduced per minute (μM H_2_O_2_/min) [16].

*Assay for (-SH) groups determination:* Total sulphydryl (-SH) groups were measured spectrophotometrically at 412 nm in phosphate buffer (0.2 mol + 2 mmol EDTA, pH 9) using 5,5-dithiobis-2-nitrobenzoic acid (DTNB, 0.01 M) (by Elman, 1959) [17]. The results are expressed as μM of SH/mL ± SEM.

*Enzyme-linked immunosorbent assay (IL-6):* The concentration of total proteins in serum samples was measured using the BCA protein assay kit (23225, Pierce BCA protein assay kit, Thermo Scientific, USA). To determine the concentration of IL-6 in the patient’s serum, the Human IL-6 Uncoated ELISA Kit (Invitrogen, 88-7066-88) was used according to the manufacturer’s protocol. Absorbance was measured at 450nm using a microplate reader (Scientific MultiskanSkyHigh Microplate Spectrophotometer, Thermo Fischer Scientific, Massachusetts, USA). After measuring the absorbance, concentrations of IL-6 were determined based on the standard curve, and the results were normalized to the protein concentrations.

### 2.3. Outcomes

**Primary Outcome:** The primary outcome of this pilot randomized controlled trial was to assess the effect of DEX on oxidative and inflammatory stress markers in pediatric patients. Biomarkers of oxidative stress (malondialdehyde, nitric oxide, glutathione, superoxide dismutase, catalase, total sulfhydryl groups) and inflammatory response (values of white blood cells, neutrophil to lymphocyte ratio, interleukin 6 and C-reactive protein) were measured before and after treatment to determine any statistically significant changes in the intervention group compared to the control group.

**Secondary Outcomes:** Secondary outcomes included the evaluation of clinically relevant parameters to assess the broader physiological impact of inhaled Dexmedetomidine. These parameters were:

- Level of sedation, measured using the Ramsay Sedation Scale;
  - Bispectral Index (BIS) values;
  - Hemodynamic parameters, including heart rate and blood pressure;
  - Respiratory function, assessed via oxygen saturation, respiratory rate;
  - Anesthetic and analgesic consumption, recorded intraoperatively and postoperatively;
  - Incidence of complications;
  - Delirium, measured using a validated pediatric delirium scale – PAED;
  - Pain, assessed via an age-appropriate pain – VAS scale;

### 2.4. Statistical analysis

Due to the exploratory nature of the study and the specific and sensitive population (children), the study is not powered for the multiple hypothesis testing and sample size is not calculated before the study. The results obtained from this trial will serve to generate prior information for new hypotheses in future trials. For that reason, we can consider these results not as confirmation that DEX is efficient, but only that DEX can be further evaluated in a confirmatory trial with sufficient sample size that achieves minimal study power of 80% using a 0.05 type I error. The statistical testing was performed in exploratory manner. Due to the vulnerability of patients, based on literature data, a minimum of 15 individuals were selected for each group, which was approved by the Ethics Committee of the health institution where the study took place [18,19]. Data are presented as count (%), means ± standard deviation or median (25^th^ - 75^th^ Percentile) depending on data type and distribution. Groups are compared using parametric (t-test) and nonparametric (Chi-square, Mann-Whitney U test) tests. All *p*-values less than 0.05 were considered significant. All data were analyzed using SPSS 29.0 (IBM Corp. Released 2023. IBM SPSS Statistics for Windows, Version 20.0. Armonk, NY: IBM Corp.) and R 3.4.2. (R Core Team (2017). R: A language and environment for statistical computing. R Foundation for Statistical Computing, Vienna, Austria. URL https://www.R-project.org/.).

## 3. Results

**Fig 1:**
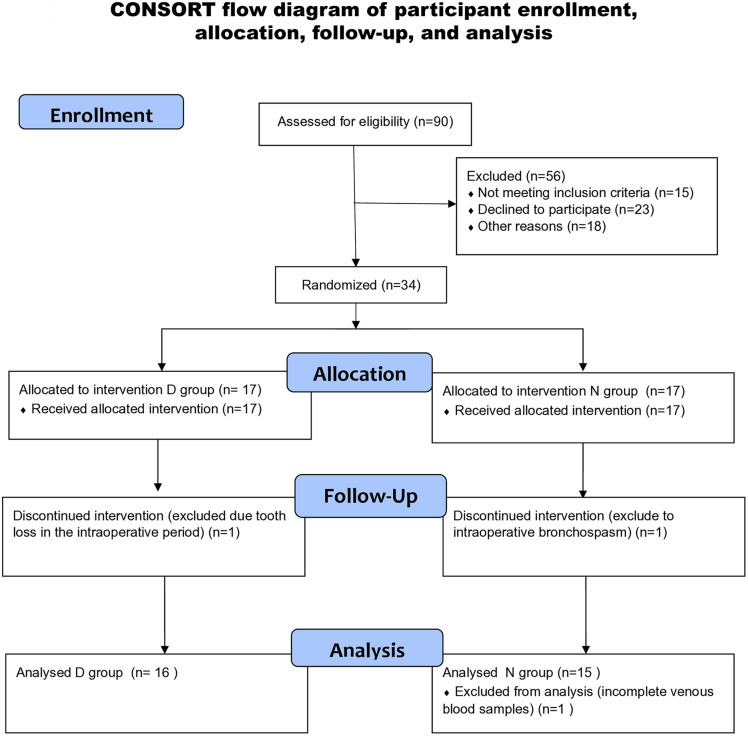
CONSORT flow diagram of participant enrollment, allocation, follow-up, and analysis.

From February to August 2023. ninety patients were assessed for eligibility, and 56 children were excluded from the study, as shown in Study flow diagram (Fig 1).

The patients were not included in the study: 15 did not meet the inclusion criteria, 23 parents refused to participate, and 18 children were younger than four years). Thirty-four patients were enrolled and randomized into intervention (D group, n = 17) and control group (N group, n = 17). One child from the intervention group (D group) was excluded from the study due to tooth loss during surgery. Two children were excluded from the control group: a child who developed intraoperative bronchospasm and a child whose blood samples were incomplete. In the final analysis of the pilot study, 16 patients participated in the D group and 15 patients in the control group. Overall, 31 patients were analyzed. Characteristics of the study population are presented in Table 1. There was no difference in age, sex, body weight and duration of surgery between the two groups. Results of oxidative stress and inflammatory response markers are presented in Tables 2 and 3. According to our results, treatment with DEX caused a statistically significant decrease in the MDA level compared to the non-treated group (p 0.014). At the same time, statistical significance was not obtained in other investigated parameters of oxidative stress and inflammatory parameters in plasma samples of pediatric patients.

**Table 1:** Characteristics of patients and duration of surgery between groups.

Parameter	Dexmedetomidine group (n = 16)	Normal saline group (n = 15)
**Age** (yr)	6.8 ± 2.2	5.8 ± 1.3
**Body weight** (kg)	24.1 ± 7.2	22.9 ± 7.2
**Sex** (M/F)	9/7	9/6
**ASA** (I/II)	16/0	15/0
**Duration of surgery** (min)	21.9 ± 4.4	22.1 ± 5.5

Values are expressed as No. (%) or mean ± SD, except ^b^ χ2 test.

ASA-American Association of Anesthesiologist; M-male; F-female.

**Table 2:** Comparison of mean values of MDA, NO, GSH, SOD, CAT and SH at various time points between two groups.

Parameter	Dexmedetomidine group (n = 16)	Normal saline group (n = 15)	p-value
**Baseline MDA** (µM/mL) ± SEM	89.25 ± 1.16	93.06 ± 1.19	<0.001
**Postoperative MDA**	88.93 ± 1.19	95.05 ± 1.58	<0.001
**Δ MDA**	−0.32	0.98 ± 1.34	0.014
**Baseline NO** (μM/mL) ± SEM	16/0	15/0	
**Postoperative NO**	21.40 ± 3.71	15.73 ± 3.60	<0.001
**Δ NO**	−0.72	−0.81 ± 2.84	0.933
**Baseline GSH** (nM/mL) ± SEM	35.26 ± 5.81	20.53 ± 3.83	<0.001
**Postoperative GSH**	38.04 ± 5.86	21.61 ± 5.00	<0.001
**Δ GSH**	2.78 ± 5.76	1.08 ± 3.29	0.338
**Baseline SOD** (U/mg protein)	1,015.24 ± 142.38	702 ± 162.29	<0.001
**Postoperative SOD**	1,109.70 ± 117.39	741.32 ± 112.31	<0.001
**Δ SOD**	94.45 ± 170.69	38.89 ± 185.62	0.4
**Baseline CAT** (U/mg protein)	6.63 ± 1.98	3.50 ± 0.98	<0.001
**Postoperative CAT**	7.14 ± 2.68	3.99 ± 1.44	<0.001
**Δ CAT**	0.51 ± 1.49	0.49 ± 0.72	0.755
**Baseline SH** (μM/mL ± SEM)	0.39 ± 0.05	0.32 ± 0.04	<0.001
**Postoperative SH**	0.40 ± 0.05	0.34 ± 0.09	0.003
**ΔSH**	0.01 ± 0.03	0.03 ± 0.05	0.539

Values are expressed as No. (%) or mean ± SD.

MDA-Malondialdehyde; NO-nitric oxide; GSH-glutathione; SOD-Superoxide dismutase; CAT-catalase; SH-total sulfhydryl groups.

**Table 3:** Comparison of mean values of white blood cells, neutrophil to lymphocyte ratio, Interleukin 6 and C-reactive protein.

Parameter	Dexmedetomidine group (n = 16)			Normal saline group (n = 15)			p-value
**WBC**_**1**_ (10^9/L) ± SD							
**Baseline WBC**_**1**_	7.63 ± 1.76			7.65 ± 1.88			0.970
**Postoperative WBC**_**2**_	13.97 ± 2.84			14.17 ± 3.72			0.870
**Δ WBC**	6.34 ± 2.74			6.51 ± 3.39			0.878
**N/L ratio**							
**Baseline N/L ratio**	1.17 ± 0.52			1.06 ± 0.39			0.514
**Postoperative N/L ratio**	5.26 ± 2.36			5.26 ± 3.29			0.999
**Δ N/L ratio**	4.09 ± 2.53			4.20 ± 3.11			0.914
**IL 6**							
**Baseline IL 6**	10.51 ± 5.24			7.65 ± 7.16			0.056
**Postoperative IL 6**	13.16 ± 7.80			8.66 ± 6.27			0.183
**ΔIL 6**	2.65 ± 9.75			1.01 ± 4.26			0.934
**CRP**	**median**	**P25**	**P75**	**median**	**P25**	**P75**	**p-value**
**Baseline CRP**_**1**_	.30	.20	.50	.50	.30	1.20	0.192
**Postoperative CRP**_**2**_	.30	.25	.50	.70	.30	1.60	0.102
**ΔCRP**	.00	−.05	.05	.10	.00	.40	0.093

Values are expressed as No. (%) or mean ± SD.

WBC-white blood cell; N/L ratio-neutrophil to lymphocyte ratio; IL6-Interleukin 6.

No statistically significant was observed in heart rate, systolic and diastolic blood pressure between groups after inhalation of Dexmedetomidine (Table 4).

**Table 4:** Comparison of mean systolic and diastolic blood pressure and heart rate at various time points between two groups.

Time points	Dexmedetomidine group (n = 16)	Normal saline group (n = 15)	p-value
**SBP** (mmHg)			
**Baseline**	110.2 ± 8.0	116 ± 9.3	0.058
**After nebulization**	103.0 ± 10.0	112.9 ± 8.5	0.006
**After intubation**	101.2 ± 9.4	108.9 ± 8.5	0.081
**After 10 min**	108.8 ± 14.5	110.5 ± 17.8	0.761
**After 15 min**	116.2 ± 11.9	122.3 ± 13.8	0.198
**SBP final**	120 ± 8.2	122.9 ± 9.7	0.411
**SBP awakening**	109.7 ± 11.5	124.8 ± 13.6	0.002
**ΔSBP 15**	5.9 ± 13	5.9 ± 17.9	0.990
**ΔSBP final**	9.9 ± 11	6.5 ± 12.9	0.424
**ΔSBP awakening**	−0.6 ± 14	8.5 ± 17.4	0.123
**DBP** (mmHg)			
**Baseline**	68.9 ± 11.3	74.3 ± 12.2	0.211
**After nebulization**	61.4 ± 12.2	72.9 ± 12.1	0.013
**After intubation**	62.4 ± 9.1	65.5 ± 11.5	0.421
**After 10 min**	70.4 ± 14.7	68.7 ± 18.3	0.776
**After 15 min**	73.9 ± 10.2	80.6 ± 14.1	0.141
**DBP final**	76.5 ± 9.9	82.3 ± 11.5	0.145
**DBP awakening**	69.4 ± 10	82.0 ± 8.6	<0.001
**ΔDBP 15**	5.1 ± 10.9	6.3 ± 16.6	0.802
**ΔDBP final**	7.6 ± 15.3	8.9 ± 15.6	0.947
**ΔDBP awakening**	0.5 ± 17.5	7.7 ± 10.7	0.179
**HR** (beats per minute)			
**Baseline**	92.7 ± 12.7	112.7 ± 21.3	0.003
**After nebulization**	89.1 ± 10.9	106.6 ± 19.1	0.004
**After intubation**	93 ± 12.9	107 ± 19.8	0.022
**After 10 min**	99.8 ± 19.4	109.9 ± 14.7	0.112
**After 15 min**	110.2 ± 18.4	120.1 ± 19.7	0.162
**HR final**	113.9 ± 17.8	128.3 ± 14.7	0.020
**HR awakening**	103.3 ± 16.9	121.7 ± 21.5	0.013
**ΔHR 15**	17.6 ± 22.3	7.4 ± 31.6	0.306
**ΔHR final**	21.2 ± 21.9	15.7 ± 22.5	0.494
**ΔHR awakening**	10.6 ± 16.0	9.0 ± 15.7	0.786

Values are expressed as No. (%) or mean ± SD.

BP – blood pressure; SBP – systolic blood pressure; DBP – diastolic blood pressure; HR- heart rate.

The groups did not differ in respiratory parameters and SpO2 values either. The patients in the treated group achieved the desired level of sedation compared to the non treated (p < 0.001). Also, there was a significant reduction in BIS values between groups 30 minutes after nebulization but not in intraoperative and postoperative time points (Tables 5, 6).

**Table 5:** Comparison of mean Ramsey scores between two groups.

Time points	Dexmedetomidine group (n = 16)		Normal saline group (n = 15)		p-value
**Baseline** **Ramsey score**	RS 1	16 (100%)	RS 1	14 (93.3%)	0.484
	RS 2	0 (0%)	RS 2	1 (6.7%)	
**After nebulization** **Ramsey score**	RS 1	0 (0%)	RS 1	12 (80%)	<0.001
	RS 2	8 (50%)	RS 2	3 (20%)	
	RS 3	5 (31.3%)	RS 3	0 (0%)	
	RS 4	3 (18%)	RS 4	0 (0%)	

Values are expressed as No. (%) or mean ± SD.

**Table 6:** Comparison of mean BIS, RR and SpO2 at various time points between two groups.

Time points	Dexmedetomidine group (n = 16)	Normal saline group (n = 15)	p-value
**BIS**			
**Baseline**	98.4 ± 1.0	98.9 ± 1.2	0.213
**After nebulization**	86.6 ± 11.6	98.1 ± 1.2	<0.001
**After intubation**	67.6 ± 15.8	72.1 ± 10.5	0.367
**After 10 min**	54.8 ± 11.9	64.3 ± 10.7	0.027
**After 15 min**	63.9 ± 6.9	66.7 ± 5.4	0.220
**BIS final**	68.3 ± 5.0	68.3 ± 5.4	0.981
**BIS awakening**	78.6 ± 6.5	81.8 ± 6.0	0.163
**Δ BIS 15 min**	−34.5 ± 6.3	−32.2 ± 5.4	0.286
**Δ BIS final**	−30.1 ± 5.0	−30.6 ± 5.9	0.786
**Δ BIS awakening**	−19.8 ± 6.3	−17.1 ± 7.0	0.257
**Respiratory rate/minute**			
**Baseline**	18.3 ± 2.9	19.7 ± 3.5	0.208
**After nebulization**	18.9 ± 3.0	19.4 ± 3.6	0.659
**After intubation**	19.6 ± 2.2	22.2 ± 5.3	0.077
**After 10 min**	20.3 ± 2.0	20.5 ± 2.0	0.833
**After 15 min**	20.9 ± 1.8	20.3 ± 1.3	0.296
**RR final**	21.0 ± 2.2	20.5 ± 1.8	0.467
**RR awakening**	21.1 ± 3.7	20.4 ± 3.6	0.621
**ΔRR 15 min**	2.7 ± 3.4	0.6 ± 3.2	0.090
**ΔRR final**	2.7 ± 3.2	0.7 ± 3.4	0.097
**ΔRR awakening**	2.8 ± 4.6	0.7 ± 2.4	0.116
**SpO2 (%)**			
**Baseline**	98.0 ± 1.3	98.7 ± 1.0	0.122
**After nebulization**	98.1 ± 1.2	98.9 ± 0.7	0.032
**After intubation**	98.8 ± 1.4	99.6 ± 0.6	0.044
**After 10 min**	99.6 ± 0.6	99.7 ± 0.6	0.853
**After 15 min**	99.8 ± 0.4	99.7 ± 0.6	0.439
**Spo**_**2**_ **final**	99.9 ± 0.3	99.5 ± 0.7	0.107
**SpO**_**2**_ **awakening**	97.2 ± 2.8	97.2 ± 3.4	0.991
**ΔSpO**_**2**_ **15 min**	1.8 ± 1.4	1.0 ± 1.3	0.097
**ΔSpO**_**2**_ **final**	1.9 ± 1.4	0.9 ± 1.1	0.033
**Δ SpO**_**2**_ **awakening**	−0.8 ± 3.3	−1.5 ± 3.5	0.598

Values are expressed as No. (%) or mean ± SD.

BP – blood pressure; SBP – systolic blood pressure; DBP – diastolic blood pressure; HR- heart rate.

There was a significant difference between groups in pain control on awakening and one hour after the operation, respectively (p = 0.024/p = 0.039), where DEX showed better effects on pain. There were no differences in observed postoperative complications between groups (Table 7)

**Table 7:** Comparison of complications and postoperative pain at various time points between two groups.

Complication time points	Dexmedetomidine group (n = 16)			Normal saline group (n = 15)			p-value
(mean ± SD)	**Yes**	**No**		**Yes**	**No**		
**During nebulization**	0 (0%)	16 (100%)		0 (0%)	15 (100%)		/
**Intraoperative**	0 (0%)	16 (100%)		0 (0%)	15 (100%)		/
**Postoperative**	7 (43.8%)	9 (56.3)		11 (73.3%)	4 (26.7%)		0.095
**Itching**	0 (0%)	16 (100%)		1 (6.7%)	14 (93.3%)		0.484
**Laryngospasm**	2 (12.5%)	14 (87.5%)		0 (0%)	15 (100%)		0.484
**Desaturation**	2 (12.5%)	14 (87.5%)		5 (33.3%)	10 (66.7%)		0.202
**Swelling of the uvula**	1 (6.3%)	15 (93.8%)		2 (6.3%)	13 (86.7%)		0.600
**Vomiting**	2 (12.5%)	14 (87.5%)		3 (20%)	12 (80%)		0.654
**Fever**	3 (18.8%)	13 (81.3%)		4 (26.7%)	11 (73.3%)		0.685
**Postoperative pain**	**median**	**P25**	**P75**	**median**	**P25**	**P75**	**p-value**
**Pain awakening**	1.0	0.5	1.0	1.0	1.0	2.0	0.024
**Pain 1.h**	1.0	0	1.5	1.0	1.0	3.0	0.039
**Pain 3.h**	2.0	1.0	4.0	2.0	1.0	4.0	0.871
**Pain 6.h**	2.5	2.0	4.0	3.0	3.0	4.0	0.113
**Pain 9.h**	3.0	1.0	4.0	2.0	1.0	3.0	0.321
**Δ Pain** (9h-awakening)	2.0	0.5	3.0	1.0	0	2.0	0.028

There was no statistically significant difference in intraoperative opioid consumption between groups (D vs. N group: fentanyl in insert: µg kg-1 mean 3.67 ± 0.78 vs. 3.94 ± 1.12, p = 0.445; Alfentanyl in µg kg-1 mean 2.02 ± 1.85 vs. 2.30 ± 2.08, p = 0.691: 66.7% of patients in the N group requested Alfentanyl five minutes before the end of the operation, in contrast to 56.2% the patients in the D group).

## 4. Discusion

The use of alpha_2_ agonist DEX is widespread in pediatric anesthesia [20–22]. Some studies showed that the use of nebulized DEX in children is a useful alternative to other routes of administration for premedication [6,23,24]. Recent studies demonstrated that DEX’s antioxidant, anti-inflammatory, and protective effects on the central nervous system, heart, lungs, kidneys, liver, and small intestine can be beneficial during anesthesia and surgery [25–27]. Malondialdehyde is a byproduct of lipid peroxidation and is commonly used as a biomarker for oxidative stress and tissue damage. A reduction in MDA levels in plasma after treatment with nebulized DEX suggests a decrease in oxidative stress in the body. The reduction of MDA levels by nebulized DEX involves several mechanisms. It suppresses the generation of reactive oxygen species (ROS) by inhibiting NADPH oxidase activity and the mitochondrial source of ROS. Additionally, it improves the redox balance by shifting thiol/disulfide ratios. Dexmedetomidine indirectly reduces oxidative stress by decreasing NF-κB activation and neutrophil recruitment and activation, thereby reducing cytokine release (TNF-α, IL-6) and the activity of oxidative enzymes. DEX decreased sympathetic tone, lowered circulating catecholamines, reduced metabolic demand, and finally, ROS production [25,26].

Results from our pilot study indicate that nebulized DEX reduces MDA important marker of oxidative stress (ΔMDA −0.32 vs. 0.98 ± 1.34, p 0.014; Table 2) but does not affect measured values of the endogenous antioxidants (tGSH and SH) nor the activity of antioxidative enzymes (SOD and CAT) compared to placebo N group. The fact that nebulized DEX can reduce the systemic inflammatory response suggests that DEX also reduces local oxidative stress in the lung tissue. The study on rabbits by Zha et al., showed that the degree of oxidative stress in the lung tissue was reduced by the use of nebulized DEX in Ventilator-induced Lung Injury via the Keap1-Nrf2-ARE Pathway [28]. Similar results were obtained in *in vitro* studies by Cui and colleagues, who demonstrated that DEX protected lung alveolar epithelial cells from apoptosis [29]. These findings are significant because various stressors affect the lungs during general anesthesia, including intraoperative mechanical ventilation, hyperoxia, hypothermia, endotracheal intubation, surgical trauma, and/or extracorporeal circulation. As a result, postoperative respiratory complications are common in the postoperative period [30]. The mediator of these complications is increased oxidative stress in the lung tissue. By administering nebulized DEX preemptively, it may be possible to reduce lung damage and decrease the risk of pulmonary complications during general anesthesia, particularly in patients undergoing mechanical ventilation in life-threatening situations or those with transplanted lungs [31]. These would be ideal for planning future clinical research. The analysis of antioxidant defense markers did not reveal any statistically significant differences between the D and N groups. This may be due to the specificity of the children’s age and the duration of surgical stress they experienced. A larger sample size of patients with extensive and long-term surgical procedures would clarify how DEX affects antioxidant defense. Other researchers, including Mottaghi et al., have reached similar conclusions in their studies of surgical patients [32]. Authors in their research have demonstrated that DEX has beneficial hemodynamic and analgesic effects when administered as an adjuvant infusion during general anesthesia for laminectomy but does not impact the value of endogenous antioxidants during surgery.

Surgical trauma, bleeding, mechanical ventilation, hyperoxia, and hypothermia during general anesthesia initiate inflammatory stress in the body. This stress involves the activation of immune, hematological and inflammatory mediators, which consequently affect stress hormones, C-reactive protein, leukocyte migration and the release of cytokines (interleukin, tumor necrosis factors, proteases, growth factors, superoxide radicals). DEX is a drug that has shown anti-inflammatory properties to reduce inflammatory markers when used perioperatively [33,34]. Meta-analyses have shown that DEX can attenuate perioperative stress and inflammation and protect surgical patients’ immune functions [1,3,34].

Our research showed no statistically significant difference in plasma values of IL6 (Δ IL 6 2.65 ± 9.75 vs 1.01 ± 4.26, p = .934), CRP (ΔCRP p = 0.093; Table 3), and neutrophils to lymphocyte ratio (Δ N/L ratio 4.09 ± 2.53 vs 4.20 ± 3.11, p = .914; Table 3) as indicators of the inflammatory response in the D and N groups, as also shown by the research of some authors [35,36]. These results may be a consequence of the small sample size of the pilot study, the immaturity of the child’s organism to promptly respond to the increased level of oxidative stress, or the dose of DEX that reaches the systemic circulation via the alveoli is not significant, so it does not cause a strong defensive response of the body The length and severity of the surgical intervention may be essential factors for developing a more vigorous inflammatory response. Tonsillotomy with adenoidectomy takes an average of about 20 minutes, and surgical trauma and bleeding are far less severe than those of tonsillectomies. However, some studies prove that DEX during general anesthesia in pediatric patients reduces inflammatory stress mediators [37]. The study by Du et al. showed that intravenous DEX was undoubtedly a better drug for reducing the inflammatory response than midazolam and normal saline in children undergoing hernia repair surgery [38].

Dexmedetomidine’s sedative and hypnotic effect is caused by binding to pre- and post-synaptic ɑ_2_A and ɑ_2_C adrenergic receptors in the brain, spinal cord and locus ceruleus. The reduced release of noradrenaline increases the release of GABA neurotransmitters [39]. Consequently, this event reduces the excitability of the sympathetic nervous system and the release of circulating catecholamines, which also provides hemodynamic stability to patients, reduces hypersalivation and agitation, and does not cause respiratory depression. Inhalation of DEX reaches the locus ceruleus faster through local brain circulation by resorption through the nasal mucosa, while most drug molecules reach the systemic circulation via the lungs. The sedative effect has been proven in many studies in children [6,40–44]. Observing the Ramsey score of sedation in both groups, we conclude that patients were better sedated in the DEX group 30 minutes after inhalation of DEX (p < 0.001). At the same time in our study, the BIS values 30 minutes after inhalation were statistically significantly different between the D and N groups (86.6 ± 11.6 vs. 98.1 ± 1.2, P < 0.001; Table 6), but not intraoperatively and postoperatively. In the study by Shafa et al., the authors did not find a statistically significant difference in perioperative BIS values with nebulized DEX at a 2 µg kg-1 dose and 4% lidocaine 4 mg kg-1 dose [45]. This is in contrast to Xiang and colleagues, who found that using DEX with sevoflurane quickly reaches lower desired BIS values [46].

DEX has biphasic effects on systemic blood pressure and causes bradycardia in children if administered rapidly intravenously or in larger doses. Hypertension is caused by the activation of the alfa_2_B receptor, while hypotension is due to centrally induced sympathicolysis and inhibition of neurotransmission in sympathetic nerves. Bradycardia is expected with DEX because the decrease in sympathetic tone activates baroreflexes and increases vagal activity [47]. Bradypnea, bradycardia and hypertension are more frequent in children than in adults [42]. The patients in our study showed hemodynamic stability, as observed by the HR, SBP and DBP values. Three children from the N group had hypertension in the awakening phase about the basic values of systolic blood pressure according to the criteria defined in the methodology (N_1._ patient 114 mmHg vs. 150 mmHg; N_8._ patient 123 mmHg vs. 148 mmHg; N_10._ patient 97 mmHg vs. 133 mmHg). One patient of group D had hypertension compared to the basal values in the awakening phase (patient D4. 100 mmHg vs. 116 mmHg). Regarding HR, no child had perioperative bradycardia. Four children from both groups had tachycardia in the awakening phase. These results did not affect the final delta values but are worth mentioning. A study conducted by Singla and his collaborators found that administering nebulized DEX at a dose of 1 µg kg-1 resulted in a more significant decrease in arterial pressure and heart rate during laryngoscopy compared to administering DEX intravenously at the same dose of 1 µg kg-1 [48]. Shrivastava et al. presented similar results in their research on the effect of Dex on the hemodynamic response during laryngoscopy [49]. Ali et al. showed that using nebulized DEX during cochlear implant implantation at a dose greater than 3 µg/kg provides satisfactory sedation but also a higher risk of hemodynamic instability, meaning that hemodynamic stability is dose-dependent [50]. DEX demonstrates greater hemodynamic stability compared to nebulized alternatives like lidocaine, ketamine, or midazolam [51,52]. By analyzing delta RR values, patients of the D and N groups showed respiratory stability in all study phases. This fact makes DEX safe for premedication in children because it does not cause respiratory depression. The results of other authors studies also showed the same data [53,54]. This fact makes DEX safe for premedication in children because it does not cause respiratory depression. The use of benzodiazepines, choral hydrate, opioids and ketamine in the premedication of children with airway obstruction and obstructive sleep apnea due to tonsillar hypertrophy can cause severe respiratory depression and desaturation, so the use of DEX seems a logical choice [55].

Perioperative complications were monitored during the study. No complications were registered after inhalation of DEX (30 minutes) and intraoperatively. After extubation, they were registered in the D group in 7 patients (43.8%) and the N group in 11 patients (73.3%), without statistical significance P = 0.095 (Table 7). The patients had itching, laryngospasm, desaturation, elevated temperature, swelling of the uvula, and vomiting (Table 7)

Dexmedetomidine given in premedication did not affect the reduction in the incidence of postoperative delirium among patients of the D and N groups (3.67 ± 0.78 vs. 3.94 ± 1.12, P = 0.991), which does not agree with the findings of Na et al. in the study where intranasal intramuscular DEX reduced the incidence of postoperative delirium in children [56,57]. Similar results were obtained by Hu, who analyzed the effect of two doses of intranasally administered DEX and showed that a dose of 1 µg kg-1was sufficient to reduce postoperative delirium. A possible explanation for our results is that the effective dose of the administered drug is higher during intranasal administration than via inhalation, which depends partly on the adaptation of the mask to the child’s face. However, many studies show that the use of DEX reduces postoperative delirium in children [58,59].

By binding DEX to alpha_2_ adrenergic receptors in the locus ceruleus, in the spinal cord and at the level of peripheral nerves, analgesia occurs as a result of blocking the transmission of nociceptive signals to the brain and reduced production of substance P and other nociceptive peptides. Some authors believe DEX’s analgetic action is exclusively local to the application site [60,61]. The level of analgesia provided by DEX alone is debatable. However, studies have shown that the need for opioids (morphine) in the postoperative period is reduced by up to 66% when DEX is used as an adjuvant drug [62–64].

In our study, the consumption of intraoperative opioids was lower in the D group than in the N group. However, no statistically significant difference was proven (D vs. N group: fentanyl in µg kg-1 mean 3.67 ± 0.78 vs. 3.94 ± 1.12, p = 0.445, i.e., alfentanil in µg kg-1 mean 2.02 ± 1.85 vs. 2.30 ± 2.08, p = 0.691). Premedication with nebulized DEX in our study showed a benefit in postoperative pain control during the awakening phase and the first hour of the child’s stay in the ENT department (pain on awakening p = 0.024; pain during the first hour of stay in the ward p = 0.039). When administered intranasally in a dose of 1 µg kg-1, effects of DEX occur after 25 minutes, and the average length of action is about 85 minutes in healthy children [65]. Higher doses of DEX usually cause a faster onset of the drug’s effect. Periods of pain relief due to the intraoperative analgesics and DEX give enough time for the child to recover fully from the effects of the anesthesia. In our study, we avoided the immediate postoperative administration of ibuprofen as an NSAID because it could affect the inflammatory response. After taking postoperative analyses of CRP, IL6 and KKS, the children received ibuprofen syrup in case of moderate and severe pain.

This pilot study has several limitations that should be acknowledged. First, the small sample size limits the statistical power to detect rare adverse events or establish definitive conclusions regarding the efficacy of DEX in pediatric surgical patients. The findings must, therefore, be interpreted with caution. Second, the limited generalizability of the ASA physical status I–II, which excludes vulnerable groups such as neonates, critically ill children, and those with complex comorbidities. As a result, these findings may not apply to the broader pediatric population or high-acuity clinical settings. Third, the lack of blinding due to resource constraints introduces a potential for observer and selection bias, particularly when assessing subjective outcomes such as sedation levels or quality of recovery. Additionally, the short duration of follow-up restricts the ability to evaluate delayed or long-term outcomes, including the potential for neurodevelopmental effects or prolonged recovery profiles. Also the duration of the surgical intervention is an important confounding factor because numerous adverse effects during more serious operations have a more significant effect on oxidative stress and inflammatory response, so the effectiveness of DEX may have been less significant in the case of a single administration of the drug in premedication. These limitations underscore the need for larger, well-controlled studies with more extended follow-up periods and more inclusive patient populations to comprehensively evaluate the efficacy of DEX in pediatric care.

## 5. Conclusion

In conclusion, the use of nebulized DEX as a premedication drug in children at a dose of 2 µg kg-1 in our pilot study showed a statistically significant reduction in levels of MDA as a marker of oxidative stress. At the same time statistical significance was not obtained in other investigated parameters of oxidative stress, endogenous antioxidants and inflammatory parameters in plasma samples of pediatric patients. Inhalation represents an alternative route of DEX administration in children and is undoubtedly beneficial because it is safe and acceptable for the pediatric patients.The sedative effect of DEX occurs up to 30 minutes after inhalation. It is accompanied by hemodynamic and respiratory stability, an advantage compared to the drugs used so far for premedication. The administration of nebulized DEX in the applied dose did not affect postoperative agitation. Also, using DEX as a premedication, patients have intraoperative hemodynamic stability, no complications, and good analgesia immediately postoperatively. These facts are important in operations where postoperative fluid intake is not time-limited.

## Supporting information

S1 File CONSORT Extension Pilot and Feasibility Trials Checklist. (DOC)

S2 File Clinical Report Form in Serbian, approved by the Research Ethics Committee. (DOCX)

S3 File Clinical Report Form in English (translation from Serbian). (DOCX)

S4 File Database of additional parameters. (XLSX)

S5 File Database of clinical and lab parameters. (XLSX)

S6 File Legend of abbreviations from database. (DOCX)
