# Supplementary material for: Effects of nebulized dexmedetomidine for premedication on the parameters of oxidative and inflammatory stress in children undergoing tonsillotomy and adenoidectomy: A pilot randomized controlled trial
Source: PLoS One. 2026 May 11;21(5):e0348763. doi: 10.1371/journal.pone.0348763 (PMC13160344; doi:10.1371/journal.pone.0348763)
Supplement: S2 File — (DOCX) [file pone.0348763.s002.docx]

**PROTOKOL ZA PRAĆENJE PACIJENTA**

**UTICAJ INHALACIONE PREMEDIKACIJE DEKSMEDETOMIDINOM NA PARAMETRE OKSIDATIVNOG I INFLAMATORNOG STRESA ZA TONZILOTOMIJE I ADENOIDEKTOMIJE U DEČIJEM UZRASTU:RANDOMIZOVANA PILOT STUDIJA**

(ISTRAŽIVAČ ASS DR VESNA STEVANOVIĆ)

Broj učesnika studije 󠇋󠇋󠇋

Broj učesnika studije: 󠇋󠇋󠇋

K**riterijumi za uključivanje u studiju**

- deca predškolskog i školskog uzrasta DA NE
- ASA I II
- tonzilotomija sa adeneidectomijom DA NE
- informisani pristanak DA NE

**Kriterijumi za neuključivanje u studiju**

- srčane bolesti DA NE
- oboljenja pluća DA NE
- alergija na lekove DA NE
- bolesti jetre DA NE
- neurološke bolesti DA NE
- gojaznost DA NE
- endokrinološka oboljenja DA NE
- bubrežne bolesti DA NE
- alergija na kikiriki i soju DA NE
- mitohondrijalna bolest DA NE
- hiperlipidemija DA NE
- dugotrajna upotreba koritkosteroida ili

antiinflamatornih lekova DA NE

- skorašnja vakcinacija DA NE

___________________________________________________________________________

󠇋 Inkluzioni /ekskluzioni kriterijumi provereni

󠇋 Saglasnost roditelja

POTPIS ISTRAŽIVAČA:

Broj učesnika studije: 󠇋󠇋󠇋

**CRF 1: PREOPERATIVNA PROCENA**

1. Pol muški ženski
2. Uzrast godine meseci
3. Telesna masa (kg)
4. Istorija prematuriteta ≤37GN
5. Iskustva sa inhalacijama?
6. Hiperreaktivnost disajnog puta?
7. Hrkanje
8. Pasivni pušači
9. Preoperativne laboratorijske analize: KKS_1_, CRP_1_, IL6_1_, vreme krvarenja, koagulacioni status
10. Mučnina tokom vožnje

Komentari:…………………………………………………………………………………….………………………………………………………………………………………………………………………………………………………………………………………………………

Broj učesnika studije: 󠇋󠇋󠇋

**CRF 2: PREMEDIKACIJA**

1. Datum operacije:
2. Topikalni anestetik 30 minuta pre venepunkcije:
3. Ramsey skor sedacije t_0_:
4. Vitalni znaci t_0_:puls TA RR SAT BIS
5. IV kanila 20G i uzimanje venskog uzorka za markere oksidativnog stresa
6. Inhalacije:

D grupa (interventna grupa):Dexmedetomidin 2µg/kg sa NaCl 0,9% do 2ml

N grupa (kontrolna grupa):0,9% NaCl 2 ml

1. Nakon 30 minuta t_1_:Ramsey skor sedacije puls TA RR SAT BIS
2. Komplikacije:
3. Komentari…………………………………………………………………………………..………………………………………………………………………………………………………………………………………………………………………………………………

Broj učesnika studije 󠇋󠇋󠇋

**CRF 3 :OPŠTA ANESTEZIJA I OPERACIJA**

1. Midazolam IV bolus 0,05mg/kg obe grupe
2. Preokisgenacija 100% kiseonikom 3 minuta
3. Indukcija u opštu anesteziju:
4. Atropin 0,01mg/kg IV
5. Fentanyl 5µg/kg IV
6. Propofol bolus 2,5-3,5mg/kg IV
7. Rokuronium 0,45-0,6mg/kg IV
8. Ventilacija 100% kiseonikom
9. Laringoskopija i intubacija
10. Vitalni znaci na 5 min T_2 do x_:puls TA SAT BIS RR
11. Održavanje anestezije:

- kiseonik 35% + vazduh 65%+ Propofol bolusi 10-20mg do vrednosti BIS-a 40-60 + Rocuronijum pp 0.15mg/kg IV + Fentanyl 1,25-2,5µg/kg IV bolus pp + Alfentanyl 5-15µg/kg pp
- dati Paracetamol 15mg/kg IV
- dati Ondrasetron 0,1mg/kg - max 4mg IV

1. 100% kiseonik ventilacija
2. Dekurarizacija Atropin 0,01mg/kg IV i Prostigmin 0.05mg/kg IV sa ekstubacijom
3. Intraoperativne komplikacije
4. Komentari ………………………………………………………………………………………………………………………………………………………………………………………………

Broj učesnika studije 󠇋󠇋󠇋

**CRF 4. POSTOPERATIVNO PRAĆENJE**

1. Vitalni znaci 15 min nakon ekstubacije (puls, TA, RR, SAT, BIS)
2. PAED skor na buđenju
3. Vitalni znaci 15 min nakon ekstubacije (puls, TA, RR, SAT, BIS)
4. Venske analize za markere oksidativnog stresa
5. VAS skor nakon 15 minuta i na odeljenju na 3 sata sledećih 12 sati
6. Komplikacije u postoperativnom periodu:
7. Nakon 6h od operacije: IL6_2_, KKS_2_ i CRP_2_
8. Komentar……………………………………………………………………………………………………………………………………………………………………………………………………………………………………………………………………………………

Broj učesnika studije 󠇋󠇋󠇋

**Ramsey skor sedacije**

| Stepen sedacije Ramsey skor | Skor |
| --- | --- |
| Budan, anksiozan | 1 |
| Budan, sarađuje, orjentisan, miran | 2 |
| Budan, odgovara na komande | 3 |
| Spava, odgovara na stimulus | 4 |
| Spava, usporeno reaguje na stimulus | 5 |
| Spava, ne odgovara na stimulus | 6 |

**KOMPLIKACIJE**

1. Hipoksemija Sat < 92%
2. Tahikardija: vrednosti veće od 20% od bazičnih vrednosti
3. Bradikardija: vrednosti manje za 20% od bazičnih vrednosti – (dodavanje atropina) ili manje od 60/min
4. Hipertenzija: veće od 20% od početnih (ili manje od 70 + 2x uzrast)
5. Hipotenzija niže za 20% od početnih (dodati efedrin)

**Terapija bola:**

1. Paracetamol 15mg/kg iv na 6 h
2. Tramadol 2mg/kg iv
3. Ibuprofen sirup 5mg/kg po

Broj učesnika studije 󠇋󠇋󠇋

| **PREMEDIKACIJA** | | | | |
| --- | --- | --- | --- | --- |
| Ime i prezime | | | M Ž | Broj istorije: |
| Uzrast: godine meseci | | | | |
| DG: | | ASA skor | | |
| TM (kg) | Topikalni anestetik u (30 min pre venepunkcije) | | | |
| Ramsey skor sedacije: 0 min (t_0_)  Bazični vitalni znaci: 0 minut (t_0_)  puls: RR: TA: SAT: BIS: | | | | |
| IV kanila: 1. venski uzorak za oksidativni stres | | | | |
| Dexmedetomidin inhalacija 30 min 2µg/kg | | | | |
| NaCl 0,9% inhalacija 30 min kontrolna grupa | | | | |
| Ramsey skor sedacije nakon 30 min od inhalacije(t_1)_  Vitalni znaci 30 min nakon otpočinjanja inhalacije (t_1_):    puls: RR: TA: SAT: BIS: | | | | |

Broj učesnika studije 󠇋󠇋󠇋

| PEAD skor | | | | | |
| --- | --- | --- | --- | --- | --- |
| ponašanje | nema uopšte | veoma malo | malo | dosta | ekstremno |
| Kontakt očima | 4 | 3 | 2 | 1 | 0 |
| Reakcije su svrsishodne | 4 | 3 | 2 | 1 | 0 |
| Svestan okoline | 4 | 3 | 2 | 1 | 0 |
| Nemiran | 0 | 1 | 2 | 3 | 4 |
| Neutešan | 0 | 1 | 2 | 3 | 4 |

| **Trajanje operacije:** | | | | | | | | | | **nakon 15 min** | |
| --- | --- | --- | --- | --- | --- | --- | --- | --- | --- | --- | --- |
| Vitalni znaci | 5' | 10' | 15' | 20' | 25' | 30' | 35' | 40' | 45' | |  |
| puls |  |  |  |  |  |  |  |  |  | |  |
| TA |  |  |  |  |  |  |  |  |  | |  |
| SAT |  |  |  |  |  |  |  |  |  | |  |
| RR |  |  |  |  |  |  |  |  |  | |  |
| BIS |  |  |  |  |  |  |  |  |  | |  |
| Fentanyl  Alfentanyl  Propofol |  |  |  |  |  |  |  |  |  | |  |
| Skor bola nakon 15 minuta (postoperativno) | | | | | | |  |  |  | |  |

Ukupan skor:

**KOMPLIKACIJE:**

1. Preoperativne
2. Intraoperativne
3. Postoperativne

Broj učesnika studije 󠇋󠇋󠇋

**POSTOPERATIVNO PRAĆENJE DETETA**


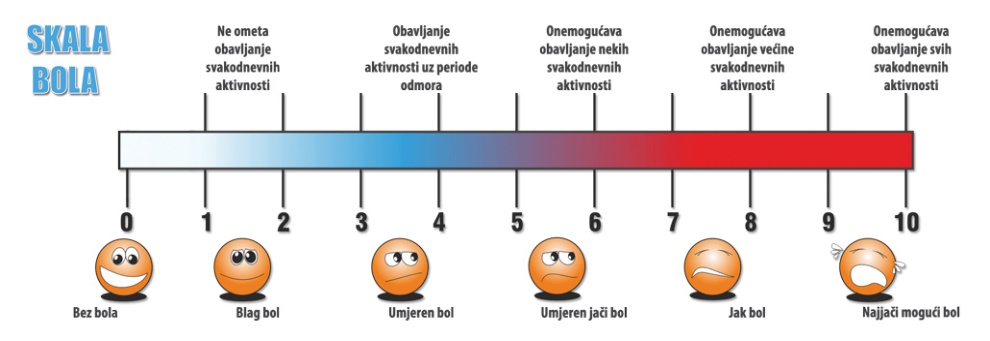


| Skor bola na 3 h  (sledećih 12h) | Analgetici  ime, doza i vreme | Komplikacije |
| --- | --- | --- |
| 1. |  |  |
| 2. |  |  |
| 3. |  |  |
| 4. |  |  |
