# Supplementary material for: Effects of nebulized dexmedetomidine for premedication on the parameters of oxidative and inflammatory stress in children undergoing tonsillotomy and adenoidectomy: A pilot randomized controlled trial
Source: PLoS One. 2026 May 11;21(5):e0348763. doi: 10.1371/journal.pone.0348763 (PMC13160344; doi:10.1371/journal.pone.0348763)
Supplement: S3 File — (DOCX) [file pone.0348763.s003.docx]

**CASE REPORT FORM**

**INFLUENCE OF INHALATION PREMEDICATION WITH DEXMEDETOMIDINE ON OXIDATIVE AND INFLAMMATORY STRESS FOR TONSILLOTOMIES AND ADENOIDECTOMY IN CHILDREN:A PILOT RANDOMIZED CONTROLLED TRAIL**

**(**RESEARCHER ASSOCIATE DR. VESNA STEVANOVIC)

number of patient󠇋󠇋󠇋

number of patient󠇋󠇋󠇋

**Inclusion criteria**

- children of preschool and school-age YES NO
- ASA I II
- tonsillotomy with adenoidectomy YES NO
- informed consent YES NO

**Exclusion criteria**

- heart disease YES NO
- lung diseases YES NO
- drug allergy YES NO
- liver disease YES NO
- neurological diseases YES NO
- obesity YES NO
- endocrinological diseases YES NO
- kidney diseases YES NO
- peanut and soy allergy YES NO
- mitochondrial disease YES NO
- hyperlipidemia YES NO
- long-term use of corticosteroids or

anti-inflammatory drugs YES NO

- recent vaccination YES NO

___________________________________________________________________________

󠇋 inclusion/exclusion criteria checked

󠇋 parental/guardians consent

SIGNATURE OF THE RESEARCHER

number of patient󠇋󠇋󠇋

**CRF 1: preoperative assessment**

1. gender male female
2. age years months
3. body weight (kg)
4. history of prematurity ≤37gn
5. experience with inhalations?
6. hyperreactivity of the respiratory tract?
7. snoring
8. passive smokers
9. preoperative laboratory analyses: KKS1, CRP1, IL61, bleeding time, coagulation status
10. seasick

Comments:…………………………………………………………………………………….……………………………………………………………………………………………………………………………………………………………………………………………………

number of patient󠇋󠇋󠇋

**CRF 2: premedication**

1. date of operation
2. topical anesthetic 30 minutes before venipuncture:
3. Ramsey sedation score t_0_
4. vital signs t_0_:pulse BP RR SAT BIS
5. IV cannula 20G and taking a venous sample for markers of oxidative stress
6. Inhalation

D group (intervention group): Dexmedetomidine 2µg/kg with NaCl 0.9% up to 2ml

N group (control group):0,9% NaCl 2 ml

1. After 30 minutes t_1_:Ramsey sedation score pulse BP RR SAT BIS
2. Complications:
3. Comments:………………………………………………………………………………….………………………………………………………………………………………………………………………………………………………………………………………………

number of patient󠇋󠇋󠇋

**CRF 3 :general anaesthesia and surgery**

1. Midazolam IV bolus 0,05mg/kg D and N group
2. preoxygenation with 100% oxygen for 3 minutes
3. induction in general anaesthesia
4. Atropine 0,01mg/kg IV
5. Fentanyl 5µg/kg IV
6. Propofol bolus 2,5-3,5mg/kg IV
7. Rokuronium 0,45-0,6mg/kg IV
8. ventilation with 100% oxygen
9. laryngoscopy and intubation
10. vital signs for 5 minutes t_2 - x_:pulse BP SAT BIS
11. maintenance of anesthesia:

- Oxygen 35% + air 65% + Propofol boluses 10-20mg up to BIS value 40-60 + Rocuronium pp 0.15mg/kg IV + Fentanyl 1.25-2.5µg/kg IV bolus pp + Alfentanyl 5-15µg /kg
- Paracetamol 15mg/kg IV
- Ondrasetron 0,1mg/kg - max 4mg IV

1. 100% Oxygen + ventilation
2. decurarization: Atropine 0,01mg/kg IV with Prostigmin 0.05mg/kg IV
3. intraoperative complications
4. Comments…………………………………………………………………………………..………………………………………………………………………………………………………………………………………………………………………………………………

number of patient󠇋󠇋󠇋

**CRF 4. postoperative period**

1. Vital signs 15 minutes after extubation (pulse, TA, RR, SAT, BIS)
2. PAED score on awakening
3. Venous analysis for markers of oxidative stress
4. VAS score after 15 minutes and in the ward for 3 hours for the next 12 hours
5. Complications in the postoperative period:
6. After 6 hours from the operation: IL62, KKS2 and CRP2
7. Comments…………………………………………………………………………………….……………………………………………………………………………………………………………………………………………………………………………………………

number of patient󠇋󠇋󠇋

**Ramsey sedation score**

| Ramsey sedation score | Score |
| --- | --- |
| Awake, anxious | 1 |
| Alert, cooperative, oriented, calm | 2 |
| Awake, responds to commands | 3 |
| Sleeps, responds to stimulus | 4 |
| Sleeps and reacts slowly to the stimulus | 5 |
| Sleep does not respond to stimulus | 6 |

**COMPLICATIONS:** definitions

1. Hypoxemia SAT < 92%

2. Tachycardia: values ​​greater than 20% of baseline values

3. Bradycardia: values ​​less than 20% of baseline values ​​- (addition of atropine IV) or less than 60/min

4. Hypertension: greater than 20% of initial (or less than 70 + 2x age)

5. Hypotension lower by 20% from the initial (add IV ephedrine)

**PAIN THERAPY**

1. Paracetamol 15mg/kg na 6 h IV
2. Tramadol 2mg/kg IV
3. Ibuprofen sirup 5mg/kg PO

number of patient󠇋󠇋󠇋

| **PREMEDICATION** | | | | |
| --- | --- | --- | --- | --- |
| Name and surname | | | M F | Patient ID |
| AGE years months | | | | |
| Diagnosis: | | ASA score | | |
| BW (kg) | Topical anesthetic in (30 min before venous puncture | | | |
| Ramsey sedation score: 0 min (t_0_)  Baseline vital signs: 0 min (t_0_)  pulse: RR: BP: SAT: BIS: | | | | |
| IV canila: 1. venous sample for markers of oxidative stress | | | | |
| Dexmedetomidin inhalation 30 min 2µg/kg - D group | | | | |
| NaCl 0,9% inhalation 30 min control group -N group | | | | |
| Ramsey sedation score 30 min after inhalation (t_1)_  Vital signs 30 min after inhalation (t_1_):    pulse : RR: BP: SAT: BIS: | | | | |

number of patient󠇋󠇋󠇋

| **PEAD score** | | | | | |
| --- | --- | --- | --- | --- | --- |
| **Description of items** | not at al | just a little | quite a bit | very much | extremely |
| The child makes eye contact with the caregiver | 4 | 3 | 2 | 1 | 0 |
| The child’s actions are purposeful | 4 | 3 | 2 | 1 | 0 |
| The child is aware of his/her surroundings | 4 | 3 | 2 | 1 | 0 |
| The child is restless | 0 | 1 | 2 | 3 | 4 |
| The child is inconsolable | 0 | 1 | 2 | 3 | 4 |

| **Operation in minutes** | | | | | | | | | | **After**  **15 min** | |
| --- | --- | --- | --- | --- | --- | --- | --- | --- | --- | --- | --- |
| Vital signs | 5' | 10' | 15' | 20' | 25' | 30' | 35' | 40' | 45' | |  |
| Heart rate |  |  |  |  |  |  |  |  |  | |  |
| BP |  |  |  |  |  |  |  |  |  | |  |
| SAT |  |  |  |  |  |  |  |  |  | |  |
| RR |  |  |  |  |  |  |  |  |  | |  |
| BIS |  |  |  |  |  |  |  |  |  | |  |
| Fentanyl  Alfentanyl  Propofol |  |  |  |  |  |  |  |  |  | |  |
| Pain score after 15 minutes (postoperative period) | | | | | | |  |  |  |  |  |

**Total**:

**COMPLICATIONS**

1. Preoperative
2. Intraoperative
3. Postoperative

number of patient󠇋󠇋󠇋


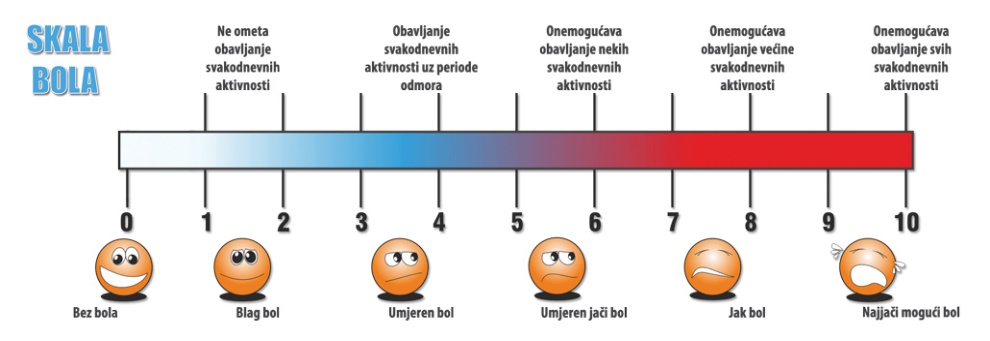


Visual Analog Scale in Serbian

| Pain score pain at three hours (next 12 hours) | Analgetics  Name, dose and time | Complications |
| --- | --- | --- |
| 1. |  |  |
| 2. |  |  |
| 3. |  |  |
| 4. |  |  |
