# Supplementary material for: Effects of nebulized dexmedetomidine for premedication on the parameters of oxidative and inflammatory stress in children undergoing tonsillotomy and adenoidectomy: A pilot randomized controlled trial
Source: PLoS One. 2026 May 11;21(5):e0348763. doi: 10.1371/journal.pone.0348763 (PMC13160344; doi:10.1371/journal.pone.0348763)
Supplement: S6 File — (DOCX) [file pone.0348763.s006.docx]

1.drug: NS normal saline (the number means the number of subjects in that group)

D – dexmedetomidine (the number means the number of subjects in that group)

2. initials: initials of the patient

3. gender: m/f

4. age: 5.11 (years, months)

5. tm: kilograms (with one decimal)

6. ASA: 1 or 2

7. RS t0 - Ramsay scale of 1-6 initial score before inhalation

8. pulse t0 - initial pulse fr/min, before inhalation

9. breath t0 - initial breathing frequency, before inhalation

10. sysTA t0 - initial systolic pressure in mmHg, before inhalation

11. dia TA t0 - initial diastolic pressure, before inhalation

12.map t0 - initial mean pressure, before inhalation

13.sat t0 – initial saturation, before inhalation

14. BIS t0 – initial BIS, before inhalation

15. RS t1 - Ramsay scale of 1-6 score 30 minutes after inhalation

16. pulse t1 - pulse fr/min 30 minutes after inhalation

17. breath t1 - breathing frequency, 30 minutes after inhalation

18. sysTA t1 - systolic pressure in mmHg, 30 minutes after inhalation

19. dia TA t1 - diastolic pressure, 30 minutes after inhalation

20. map t1 - mean pressure, 30 minutes after inhalation

21. sat t1 –saturation, 30 minutes after inhalation

22. BIS t1 – initial BIS, 30 minutes after inhalation

23.pulse t2 –pulse, 5. minutes of anesthesia (laryngoscopy)

24. breath t2 - breathing frequency, 5. minutes of anesthesia (laryngoscopy)

25. sysTA t2 - systolic pressure in mmHg, 5. minutes of anesthesia (laryngoscopy)

26. dia TA t2 - diastolic pressure, 5. minutes of anesthesia (laryngoscopy)

27. map t2 - mean pressure, 5. minutes of anesthesia (laryngoscopy)

28. sat t2 –saturation, 5. minutes of anesthesia (laryngoscopy)

29. BIS t2 – initial BIS, 5. minutes of operation (laryngoscopy)

30. pulse t3 – pulse, 10th minute of surgery

31. breath t3 - breathing frequency, 10th minute of surgery

32. sysTA t3 - systolic pressure in mmHg, 10th minute of surgery

33. dia TA t3 - diastolic pressure, 10th minute of surgery

34. map t3 - mean pressure, 10th minute of surgery

35. sat t3 –saturation, 10th minute of surgery

36. BIS t3 – initial BIS, 10th minute of surgery

37. pulse t4 – pulse 15th minute of surgery

38.breath t4 - breathing frequency, 15th minute of surgery

39. sysTA t4 - systolic pressure in mmHg, 15th minute of surgery

40. dia TA t4 - diastolic pressure, 15th minute of surgery

41. map t4 - mean pressure, 15th minute of surgery

42. sat t4 –saturation, 15th minute of surgery

43. BIS t4 –BIS, 15th minute of surgery

44.pulse t5 – pulse , 20th minute of surgery

45.breath t4 - breathing frequency, 20th minute of surgery

46. sysTA t4 - systolic pressure in mmHg, 20th minute of surgery

47. dia TA t4 - diastolic pressure, 20th minute of surgery

48. map t4 - mean pressure, 20th minute of surgery

49. sat t4 –saturation, 20th minute of surgery

50. BIS t4 –BIS, 20th minute of surgery

51. pulse t6 – pulse, 25th minute of surgery

52.breath t6 - breathing frequency, 25th minute of surgery

53. sysTA t6 - systolic pressure in mmHg, 25th minute of surgery

54. dia TA t6 - diastolic pressure, 25th minute of surgery

55. map t6 - mean pressure, 25th minute of surgery

56. sat t6–saturation, 25th minute of surgery

57. BIS t6 –BIS, 25th minute of surgery

58. pulse t7 – pulse, 30th minute of surgery

59.breath t7 - breathing frequency, 30th minute of surgery

60. sysTA t7 - systolic pressure in mmHg, 30th minute of surgery

61. dia TA t7 - diastolic pressure, 30th minute of surgery

62. map t7 - mean pressure, 30th minute of surgery

63. sat t7–saturation, 30th minute of surgery

64. BIS t7 –BIS, 30th minute of surgery

65. pulse t8 – pulse, 35th minute of surgery

66. breath t8 - breathing frequency, 35th minute of surgery

67. sysTA t8 - systolic pressure in mmHg, 35th minute of surgery

68. dia TA t8 - diastolic pressure, 35th minute of surgery

69. map t8 - mean pressure, 35th minute of surgery

70. sat t8–saturation, 35th minute of surgery

71. BIS t8 –BIS, 35th minute of surgery

72. operation - duration of the operation in minutes and seconds

73.pulseAW- puls upon awakening, after 15 minutes

74.breath AW – respiratory rate in minutes upon awakening, after 15 minutes

75.sys TA AW – systolic pressure upon awakening, after 15 minutes

76.dia TA AW – diastolic pressure upon awakening, after 15 minutes

77.map AW – mean pressure on awakening, after 15 minutes

78.sat AW – saturation upon awakening, after 15 minutes

79.BIS AW – BIS on awakening, after 15 minutes

80.PEAD - delirium score on awakening after 15 minutes

81.Pain AW - numerical pain scale 1-10 upon awakening after 15 minutes

82. fenta/kg- µg/kg fentanyl for surgery

83. alfenta/kg- µg /kg alfentanyl for surgery

84. pro/kg – total amount in mg of propofol consumed for surgery

85.pain 1- numerical pain scale 1-10 first measurement in the ward

86.pain 2-numerical pain scale 1-10 first measurement after 3 hours

87.pain 3 - numerical pain scale 1-10 first measurement after 6 hours

88. pain 4 - numerical pain scale 1-10 first measurement after 9 hours

89. preop K - complications during inhalation: yes/no

90. io K – intraoperative complications: yes/no

91. postopK- postoperative complications: yes/no

92. itching: yes/no

93. laryngospasm. yes/no

94. desaturation: yes/no

95. swelling of the uvula: yes/no

96. vomiting: yes/no

97. temp: elevated temperature: yes/no

98. WBC1: preoperative leukocyte count

99. N/L 1: the ratio of the absolute number of neutrophils and lymphocytes preoperatively

100. CRP 1: preoperative CRP

101. WBC2: leukocyte count postoperatively

102. N/L2: the ratio of the absolute number of neutrophils and lymphocytes postoperatively

103. CRP 2: CRP postoperatively

104. hosp: length of hospitalization in days

105. IL6 1 - interleukin 6 - first sample, before inhalation (pg/mg protein)

106. IL6 4- interleukin 6 - second sample, after surgery (pg/mg protein)

107. protein 1

108 .protein 4

109. MDA 1 – malondialdehyde (uM/mg prot) before inhalation

110. MDA 4 - malondialdehyde (uM/mg prot) after surgery

111. NO 1 (uM/mg prot) before inhalation

112. NO 4 (uM/mg prot) after surgery

113. GSH 1 (nM/mg prot) before inhalation

114. GSH 4 (nM/mg prot) after surgery

115. SOD 1 (U/mg prot) before inhalation

116. SOD 4 (U/mg prot) after surgery

117. CAT 1 (U/mg prot) before inhalation

118. CAT 4 (U/mg prot) after surgery

119. SH 1 (uM SH/mg prot) before inhalation

120. SH 4 (uM SH/mg prot) after surgery
